# Association between low body temperature on admission and in-hospital mortality according to body mass index categories of patients with sepsis

**DOI:** 10.1097/MD.0000000000031657

**Published:** 2022-11-04

**Authors:** Yuta Ito, Daisuke Kudo, Shigeki Kushimoto

**Affiliations:** a Department of Surgery, Osaki Citizen Hospital, Osaki, Japan; b Division of Emergency and Critical Care Medicine, Tohoku University Graduate School of Medicine, Sendai, Japan.

**Keywords:** body mass index, body temperature, critical illness, interaction, observational study

## Abstract

Hypothermia has been shown to be associated with a high mortality rate among patients with sepsis. However, the relationship between hypothermia and body mass index (BMI) with respect to mortality remains to be elucidated. We conducted this study to assess the association between hypothermia and survival outcomes of patients with sepsis according to BMI categories. This secondary analysis of a prospective cohort study enrolled 1184 patients (aged ≥ 16 years) with sepsis hospitalized in 59 intensive care units in Japan. Patients were divided into 3 BMI categories (<18.5 [low], 18.5–24.9 [normal], >24.9 [high] kg/m^2^) and 2 body temperature (36 °C and ≥ 36 °C) groups. The primary outcome was in-hospital mortality rate. Associations between hypothermia and BMI categories with respect to in-hospital mortality were evaluated using multivariate logistic regression analysis. Of the 1089 patients, 223, 612, and 254 had low, normal, and high BMI values, respectively. Patients with body temperature < 36 °C (hypothermia) had a higher in-hospital mortality rate than that had by those without hypothermia in the normal BMI group (25/63, 39.7% vs. 107/549, 19.5%); however, this was not true for patients in the low or high BMI groups. A significant interaction was observed between hypothermia and normal BMI for in-hospital mortality (odds ratio, 1.56; 95% confidence interval, 1.00–3.41; *P* value for interaction = .04); however, such an interaction was not found between hypothermia and low or high BMIs. Patients with sepsis and hypothermia in the normal BMI subgroup may have a higher mortality risk than that of those in the low or high BMI subgroups and, therefore, require more attention.

## 1. Introduction

Sepsis involves physiological, pathological, and biochemical abnormalities caused by an aberrant host reaction to infection, resulting in life-threatening organ dysfunction.^[1]^ Additionally, the mortality rate among patients with sepsis remains high (up to 40%).^[2]^

Previous studies have shown that hypothermia is associated with a high mortality rate among patients with sepsis.^[3–6]^ Although a high body mass index (BMI) is associated with poor life expectancy,^[7]^ improved survival has been observed with obesity in critical illnesses such as heart failure, acute coronary syndrome, and chronic obstructive pulmonary disease.^[8–10]^ Recently, decreased mortality among patients with a high BMI^[11]^ and increased mortality among those with a low BMI were also observed in sepsis cases.^[2]^ However, normal body temperature is related to BMI.^[12]^ People with a low BMI have less adipose tissue,^[13]^ which contributes to thermogenesis and immunologic function.^[14,15]^ Thus, patients with sepsis and a low BMI may be prone to hypothermia and high mortality.

However, the interaction of hypothermia with a higher mortality rate based on the BMI of patients with sepsis has not yet been elucidated. This study aimed to clarify the association between initial hypothermia and survival outcomes of patients with sepsis according to BMI categories.

## 2. Methods

### 2.1. Design and setting

This study was a secondary analysis of data collected from the Focused Outcome Research on Emergency Care for Acute Respiratory Distress Syndrome, Sepsis, and Trauma (FORECAST) sepsis study. The FORECAST sepsis study, a multicenter, prospective cohort of patients with sepsis enrolled at 59 intensive care units (ICUs), conducted between January 2016 and March 2017, describes the incidence, clinical characteristics, and evolving management of sepsis in Japan.^[16]^ It was registered with the University Hospital Medical Information Network Clinical Trials Registry (UMIN-CTR ID: UMIN000019742), and the study protocol was reviewed and approved by the review board of all participating institutes. The requirement for informed consent was waived by the ethics committee/institutional review board because this was an observational study without any intervention.

### 2.2. Participants and data collection

The FORECAST sepsis study registered patients with severe sepsis as defined in the Sepsis-2 definition^[17]^ since it was planned before the publication of the Sepsis-3 definition.^[1]^ All patients registered in the FORECAST sepsis study were eligible for this secondary analysis. However, they were excluded if there were any missing data on body temperature, BMI at admission, or survival at discharge. We extracted data on patient demographics, comorbidities, suspected sites of infection, organ dysfunction, and sepsis-related severity scores from the FORECAST database with the assistance of FORECAST investigators. Data on the duration of mechanical ventilation and ICU stay, 28-day and in-hospital outcomes, and disposition after discharge (that is, discharge to home or transfer to other hospitals), were also extracted.

### 2.3. Group assignment

Patients were divided into 3 groups according to the BMI categories defined by the World Health Organization (Geneva) (BMI < 18.5 [low], 18.5–24.9 [normal], and > 24.9 [high] kg/m^2^), and then further classified into 2 groups according to their core temperature (<36 °C [hypothermia] or ≥ 36 °C) measured at the time of ICU admission.^[18]^

### 2.4. Outcome measurements and definitions

The main outcome measure was in-hospital mortality rate, while the secondary outcomes were 28-day mortality, disposition after discharge, and ICU- and ventilator-free days (VFDs).

VFDs were defined as the number of days for which a patient could breathe without ventilator support during the initial 28 days after enrollment in the study. The number of VFDs for patients who died during the study period was 0. The number of ICU-free days was similarly calculated. Severe sepsis was defined as a diagnosis of or suspected new-onset infection based on the history of the present illness, with at least 2 systemic inflammatory response syndrome criteria^[12]^ and at least 1 organ dysfunction criterion met.^[17]^ In addition, septic shock was defined as a condition in which hypotension due to sepsis persists despite appropriate initial fluid resuscitation according to the Sepsis-2 criteria.^[17]^ The Charlson comorbidity index was classified into the 4 previously defined grades: 0 (none), 1–2 (low), 3–4 (moderate), and ≥ 5 points (high).^[19]^

### 2.5. Statistical analysis

Descriptive statistics included proportions for categorical variables and medians (interquartile range) for continuous variables with skewed distribution. As the amount of missing data was low, no assumptions were made. Categorical variables were compared using Fisher’s exact test or the chi-square test. Additionally, the Kruskal–Wallis one-way analysis of variance was used to compare values among multiple groups. We evaluated associations between BMI category and hypothermia to examine the heterogeneity of the effect of hypothermia on in-hospital mortality across BMI categories. In addition, we performed a multivariable logistic regression analysis, including the interaction between each BMI category and hypothermia adjusted for age, Charlson comorbidity index, and Sequential Organ Failure Assessment (SOFA) score. A similar analysis of BMI as a continuous variable was performed. We also assessed the association between in-hospital mortality and hypothermia for all patients using a logistic regression model adjusted for the same variables and BMI categories. Statistical analyses were performed using the JMP Pro Version 15 software (SAS Institute Japan Ltd., Tokyo, Japan).

## 3. Results

**Figure 1.**
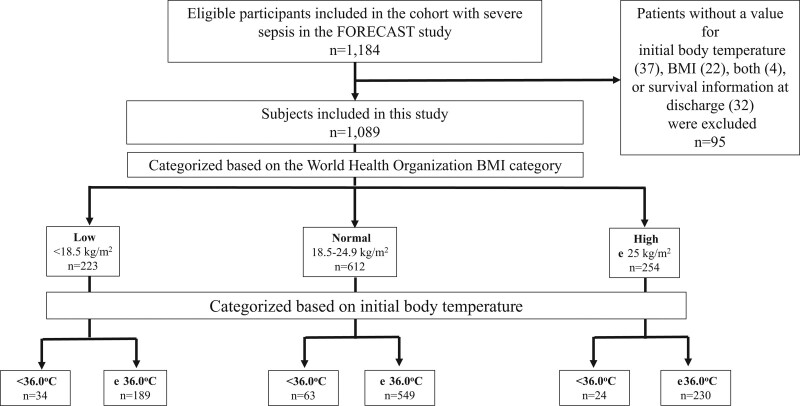
Flowchart of patient selection. BMI = body mass index.

Of the 1184 patients with severe sepsis included in the FORECAST study, 95 with missing data on body temperature, BMI at admission, or survival information at hospital discharge were excluded; therefore, we analyzed the data of 1089 patients in the current sub-analysis. Of these patients, 223, 612, and 254 were categorized as having low, normal, and high BMI, respectively. Furthermore, the patients were divided into 2 groups (with and without hypothermia) (Fig. 1).

### 3.1. Baseline characteristics and outcomes

Patient characteristics for each BMI and body temperature group are shown in Table 1. The patients’ median age was 73 (64–81) years; 61.0% of the patients were men, and the median BMI was 21.7 (19.0–24.7) kg/m^2^. The most common sites of infection were the lungs (31.3%), abdomen (26.1%), and urinary tract (18.6%). The sites used for temperature measurement were the bladder, 559; axilla, 416; and tympanic membrane, 68. Vasopressors were administered in 677 patients. The most frequently used vasopressor was noradrenaline (see Table 3, Supplementary Content, http://links.lww.com/MD/H871, which demonstrates body temperature measurement sites and the types of vasopressors used in each group). Additionally, the median Acute Physiology and Chronic Health Evaluation (APACHE) II and SOFA scores on the day of admission were 22 (17–29) and 9 (6–11), respectively. More than two-thirds of all patients had preexisting comorbidities, evaluated using the Charlson comorbidity index. The occurrence of septic shock, acute kidney injury, and high SOFA and APACHE II scores on arrival was higher for patients with hypothermia than for those without hypothermia in the low and normal BMI groups. We also showed patient characteristics according to BMI categories with or without low body temperature (see Tables 4 and 5, Supplemental Content, http://links.lww.com/MD/H872, which illustrate the patient characteristics according to BMI categories and patient characteristics with or without low body temperature, respectively).

**Table 1:** Characteristics of patients with and without hypothermia according to the body mass index (BMI) category on admission.

	All	Low BMI (n = 223)			Normal BMI (n = 612)			High BMI (n = 254))		
		<36.0 °C (n = 34)	≥36.0 °C (n = 189)	*P* value	<36.0 °C (n = 63)	≥36.0 °C (n = 549)	*P* value	<36.0 °C (n = 24)	≥36.0 °C (n = 230)	*P* value
Age, yr	73 (64–81)	76 (67–85)	75 (66–83)	.3197	75 (70–84)	73 (64–81)	.0039	69 (64–80)	69 (61–79)	.79
Male sex, n, %	664, 61.0%	15, 44.1%	109, 57.7%	.1431	35, 55.6%	352, 64.1%	.1819	16, 66.7%	137, 59.6%	.46
BMI, kg/m^2^	21.7 (19–24.7)	17.1 (15.9–18.1)	16.8 (15.1–17.8)	.3434	21.4 (19.6–22.6)	21.4 (19.6–22.6)	.5105	28 (26–31.5)	27 (25.8–30.6)	.32
Charlson Comorbidity Index, n, %				.588			.0352			.43
0	360, 33.1%	12, 35.3%	66, 34.9%		15, 23.8%	182, 33.2%		6, 25.0%	79, 34.4%	
1-2	502, 46.1%	16, 47.1%	88, 46.6%		29, 46.0%	261, 47.5%		14, 58.3%	94, 40.9%	
3-4	165, 15.2%	6, 17.7%	26, 13.8%		16, 25.4%	69, 12.6%		3, 12.5%	45, 19.6%	
>4	62, 5.7%		9, 4.8%		3, 4.8%	37, 6.7%		1, 4.2%	12, 5.2%	
Activities of daily living: inactive, n, %	830, 76.2%	23, 67.7%	118, 62.4%	.5617	53, 84.1%	426, 77.6%	.4773	21, 87.5%	189, 82.2%	.51
Suspected site of infection, n, %	1089	34	189	.7055	63	549	.4118	24	230	.60
Lung	341, 31.3%	11, 32.4%	72, 38.1%		20, 31.8%	168, 30.6%		10, 41.7%	60, 26.1%	
Abdomen	284, 26.1%	11, 32.4%	48, 25.4%		20, 31.8%	150, 27.3%		3, 12.5%	52, 22.6%	
Urinary tract	202, 18.6%	7, 20.6%	39, 20.6%		10, 14.4%	91, 16.6%		3, 12.5%	52, 22.6%	
Soft tissue	106, 9.73%		10, 5.29%		2, 3.17%	58, 10.6%		5, 20.8%	31, 13.5%	
Other	156, 14.3%	5, 14.7%	20, 10.6%		11, 17.5%	82, 14.9%		3, 12.5%	35, 15.2%	
Positivity of blood cultures, n, %	629, 57.8%	18, 52.9%	118, 62.4%	.2962	34, 54.0%	313, 57.0%	.6459	10, 41.7%	136, 59.1%	.10
Septic shock, n, %	698, 62.3%	28, 82.4%	119, 63.0%	.0281	50, 79.4%	332, 60.5%	.0034	13, 54.2%	132, 57.4%	.76
Hypotension, n, %	543, 49.9%	24, 70.6%	96, 50.8%	.0331	43, 68.3%	279, 50.8%	.0087	6, 25.0%	95, 41.3%	.12
Hyperlactatemia, n, %	724, 66.5%	26, 76.5%	126, 66.7%	.2586	48, 76.2%	360, 65.6%	.0904	14, 58.3%	150, 65.2%	.50
Acute kidney injury, n, %	408, 37.5%	18, 52.9%	50, 26.5%	.002	36, 57.1%	181, 33.0%	.0001	14, 58.3%	109, 47.4%	.31
Acute lung injury, n, %	141, 14.4%	3, 8.8%	12, 6.4%	.5959	13, 20.6%	69, 12.6%	.075	2, 8.3%	42, 18.3%	.22
APACHE2 score	22 (17–29)	28 (19–34)	22 (17–29)	.0112	28 (21–35)	22 (17–28)	.0002	27 (14–35)	22 (16–30)	.09
SOFA score	9 (6–11)	10 (7–13)	9 (5–11)	.0076	11 (8–13)	8 (5–11)	<.0001	10 (6–12)	9 (5–12)	.37

Number of patients with missing data: activities of daily living, n = 1; positivity of blood cultures, n = 6; SOFA score n = 158; APACHE II score, n = 126.

APACHE = acute physiology and chronic health evaluation, BMI = body mass index, SOFA = sequential organ failure assessment.

**Table 3:** Odds ratios of the interaction for in-hospital mortality.

Variables	*P*-value for interaction
Low BMI × BT < 36.0	.0566
Normal BMI × BT < 36.0	.0397
High BMI × BT < 36.0	.6133

BMI = body mass index, BT = body temperature.

In-hospital mortality rates were 38/121 (31.4%) and 207/968 (21.4%) among patients with and without hypothermia, respectively (see Table 1, Supplemental Content, http://links.lww.com/MD/H869, which demonstrates the clinical outcomes of patients with and without hypothermia). Among patients with normal BMI, in-hospital and 28-day mortality rates, ICU-free days, and VFDs were significantly different between the groups with and without hypothermia (Table 2). In addition, in the groups with and without hypothermia, in-hospital mortality rates were 25/63 (39.7%) and 107/549 (19.5%), respectively (*P* = .002), among those with normal BMI. Importantly, among patients with low and high BMI, the outcomes, except for ICU-free days, were similar between the body temperature groups.

**Table 2:** Clinical outcomes in patients with and without hypothermia classified by body mass index (BMI).

Outcomes	All patients	Low BMI (n = 223)			Normal BMI (n = 612)			High BMI (n = 254)		
		<36.0 °C	≥36.0 °C	*P* value	<36.0 °C	≥36.0 °C	*P* value	<36.0 °C	≥36.0 °C	*P* value
	(n = 1089)	(n = 34)	(n = 189)		(n = 63)	(n = 549)		(n = 24)	(n = 230)	
In-hospital mortality	245/1089, 22.5%	7/34, 20.6%	47/189, 24.9%	.586	25/63, 39.7%	107/549, 19.5%	.0002	6/24, 25.0%	53/230, 23.0%	.83
28-d mortality	196/1080, 18.1%	7/34, 20.6%	38/189, 20.1%	.149	20/63, 31.8%	82/549, 14.9%	.0024	5/24, 20.8%	44/230, 19.1%	.89
Survivor dispositions	(n = 844)	(n = 27)	(n = 112)	.132	(n = 38)	(n = 442)	<.0001	(n = 18)	(n = 177)	.22
Home (n, %)	309, 36.6%	4, 14.8%	46, 24.3 %		7, 18.4%	171, 38.7%		4, 22.2%	77, 43.5%	
Transfer (n, %)	535, 63.4%	23, 85.2%	96, 50.8%		31, 81.6%	271, 61.3%		14, 77.8%	100, 56.5%	
ICU-free days	20 (12–24)	18 (11–23)	20 (13–25)	.293	16 (0–23)	19 (10–24)	.02	13 (7–20)	20 (12–24)	.03
Ventilator-free days	24 (18–28)	21 (0–28)	22 (0-28)	.621	13 (0–25)	21 (1–28)	.0011	17 (0–22.75)	21 (0–28)	.18

Number of patients with missing data: 28-d mortality, n = 9; survivor dispositions, n = 245; ICU-free days, n = 205; ventilator-free days, n = 9.

BMI = body mass index, ICU = intensive care unit.

### 3.2. Interaction between hypothermia and BMI category for in-hospital mortality

**Figure 2.**
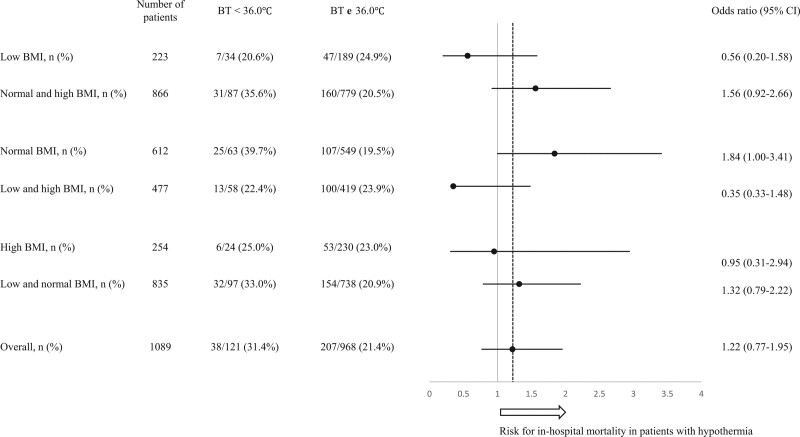
Odds ratio and *P* value for interaction between body mass index categories and hypothermia with respect to in-hospital mortality. The ORs of hypothermia for in-hospital mortality in each body mass index category are shown. ORs and 95% confidence intervals (CIs) were calculated using a multivariate logistic regression model. Black circles indicate OR, while horizontal bars indicate 95% CIs. The dotted vertical line indicates the OR of hypothermia for in-hospital mortality. Low BMI, <18.5 kg/m^2^; normal BMI, 18.5–24.9 kg/m^2^; high BMI ≥ 25 kg/m^2^. BMI = body mass index, BT = body temperature, OR = odds ratio.

Although a significant interaction was observed between normal BMI and hypothermia with respect to in-hospital mortality (odds ratio [OR] 1.84, 95% confidence interval [CI] 1.00–3.41, *P* for interaction = .04) after adjusting for age, Charlson comorbidity index, and SOFA score, this was not true between hypothermia and low/high BMI (Fig. 2, Table 3). In addition, hypothermia alone was not significantly associated with higher mortality rates (see Table 2, Supplemental Content, http://links.lww.com/MD/H870, which demonstrates the odds ratios of factors contributing to worse in-hospital mortality). Similarly, BMI alone, as a continuous variable, was not significantly associated with higher mortality rates (see Table 6, Supplemental Content, http://links.lww.com/MD/H873, which demonstrates odds ratios of factors of worse in-hospital mortality). Considering analysis of BMI as a continuous variable, the interaction was still significant in the normal body temperature group (see Table 7, Supplemental Content, http://links.lww.com/MD/H874, which demonstrates *P*-value for the interaction for in-hospital mortality).

## 4. Discussion

We found that hypothermia in patients with sepsis was associated with higher in-hospital mortality rates in the normal BMI group; however, this was not observed in the low and high BMI groups. To the best of our knowledge, this is the first study to examine the interaction between low body temperature on admission and BMI.

Previous studies have indicated that hypothermia is associated with an increased mortality risk in patients with sepsis.^[4,6,20–22]^ Patients with hypothermia had significantly higher disseminated intravascular coagulation rates, SOFA and APACHE II scores, and 28-day and in-hospital mortality rates than those had by patients without hypothermia in a large observational study of patients with sepsis.^[23]^ Furthermore, metabolic and immunologic dysfunctions induced by hypothermia^[24–26]^ lead to a higher mortality rate among patients with sepsis than among those without.

A high BMI and various comorbidities are associated with an increased risk of death.^[7]^ However, in critical illnesses such as acute coronary syndrome and chronic obstructive pulmonary disease, a high BMI is associated with lower mortality than a low or normal BMI.^[9,10]^ This phenomenon is referred to as the obesity paradox.^[8–10]^ Similarly, in patients with sepsis, a high BMI is associated with lower mortality.^[11,27]^ Conversely, several reports have shown that a low BMI is associated with increased mortality in patients with sepsis.^[28,29]^ Patients with a low BMI have less adipose tissue,^[13]^ which is involved in immunologic functions and thermogenesis.^[14,15]^ Therefore, patients with a low BMI may have weak immunity and low thermogenetic capacity. Based on the results of previous reports on hypothermia and BMI of patients with sepsis and amount of adipose tissue, we hypothesized that patients with sepsis with hypothermia and a low BMI would experience lower mortality rates. However, hypothermia was associated with higher mortality among patients with a normal BMI than among those with a low or high BMI.

Studies showing an association between hypothermia and increased risk of mortality among patients with sepsis did not use BMI categories as a covariate in the multivariate analysis. However, in our study, hypothermia was not associated with higher in-hospital mortality after adjusting for several covariates, including BMI categories, among all patients (see Table 2, Supplementary Content, which demonstrates the odds ratios of factors contributing to worse in-hospital mortality). Thus, the effect of hypothermia on mortality among patients with sepsis may differ according to BMI categories.

This study had some limitations. First, the number of patients with a low BMI and hypothermia was small. Therefore, random errors may occur in estimating the effect of hypothermia on patients with a low BMI. Second, since the method for measuring body temperature was not predefined across institutes, some patients may have been categorized into different body temperature groups if the body temperatures were measured using different body parts or tools. Third, there were no data on some factors related to body temperature, such as season, time spent outdoors, use of antipyretic drugs, and antibiotic treatment before admission. Fourth, we obtained body temperature data at a one-time point on admission. Fifth, almost all the participants in the cohort study were Japanese (99.1%, 1079/1089); therefore, the results may not be applicable to patients from other races. Finally, we could only include a limited number of explanatory variables in the multivariate analysis because of the small number of patients who died.

In conclusion, hypothermia in patients with sepsis was associated with in-hospital mortality in the normal BMI group but not in the low/high BMI groups. This subgroup may require more attention and special consideration because of the higher mortality risk to avoid a delay in treatment and management.

## Acknowledgments

We thank the core investigators of the FORECAST sepsis study for providing the dataset and Tohoku Kyuikai for financial support.

## Author contributions

**Conceptualization:** Yuta Ito.

**Data curation:** Yuta Ito, Daisuke Kudo.

**Formal analysis:** Yuta Ito.

**Funding acquisition:** Shigeki Kushimoto.

**Investigation:** Yuta Ito.

**Methodology:** Yuta Ito.

**Project administration:** Yuta Ito.

**Resources:** Yuta Ito.

**Software:** Yuta Ito.

**Supervision:** Shigeki Kushimoto.

**Visualization:** Yuta Ito.

**Writing—review and editing:** Daisuke Kudo.

**Writing—original draft:** Yuta Ito.

## Supplementary Material


