# Supplementary material for: Association between low body temperature on admission and in-hospital mortality according to body mass index categories of patients with sepsis
Source: Medicine (Baltimore). 2022 Nov 4;101(44):e31657. doi: 10.1097/MD.0000000000031657 (PMC9646569; doi:10.1097/MD.0000000000031657)
Supplement: Supplementary file 1 [file medi-101-e31657-s001.pdf]

Supplemental Table 1. Clinical outcomes in patients with and without hypothermia

Outcomes	All patients (n=1089)	＜36.0 °C (n=121)	≥36.0 °C (n=968)	P value
In-hospital mortality rate	245/1089, 22.5%	38, 31.4%	207, 21.4%	0.02
28-day mortality	196/1080, 18.1%	32/120, 26.7%	164/959, 17.1%	0.05
Survivor dispositions	(n=844)	(n=83)	(n=761)	<0.0001
Home (n, %)	309, 36.6%	15, 18.1%	294, 38.6 %	
Transfer (n, %)	535, 63.4%	68, 81.9%	467, 61.4%	
ICU-free days	20 (12–24)	15.5 (8–22.75)	20 (11–24)	0.0027
Ventilator-free days	24 (18–28)	17 (0–25)	21 (0–28)	0.0016

Number of patients with missing data: 28-day mortality, n=9; Survivor dispositions, n=245; ICU-free days, n=205; Ventilator-free days, n=9

ICU, intensive care unit
