# Supplementary material for: Association between low body temperature on admission and in-hospital mortality according to body mass index categories of patients with sepsis
Source: Medicine (Baltimore). 2022 Nov 4;101(44):e31657. doi: 10.1097/MD.0000000000031657 (PMC9646569; doi:10.1097/MD.0000000000031657)
Supplement: Supplementary file 2 [file medi-101-e31657-s002.pdf]

Supplemental Table 2. Odds ratios of factors of worse in-hospital mortality

**Variables**	Odds ratio	95% CI	P value
<36 °C	1.222	0.765–1.953	0.40
Age	1.015	1.002–1.028	0.02
Charlson Comorbidity Index	1.223	1.118–1.339	<.0001
SOFA score	1.178	1.126–1.232	<.0001
BMI low vs. normal	1.154	0.758–1.756	0.50
BMI normal vs. high	0.917	0.615–1.367	0.67
BMI high vs. low	0.945	0.577–1.550	0.82

BMI, body mass index; CI, confidence interval; SOFA, Sequential Organ Failure Assessment
