# Supplementary material for: Association between low body temperature on admission and in-hospital mortality according to body mass index categories of patients with sepsis
Source: Medicine (Baltimore). 2022 Nov 4;101(44):e31657. doi: 10.1097/MD.0000000000031657 (PMC9646569; doi:10.1097/MD.0000000000031657)
Supplement: Supplementary file 3 [file medi-101-e31657-s003.pdf]

Supplemental Table 3. Body temperature measuremen sites and the types of vasopressors according to BMI categories.

	All	Low BMI (n-223)		Normal BMI (n=612)		High BMI (n=254))	
		<36.0 ° C (n=34)	≥36.0 ° C (n=189)	<36.0 ° C (n=63)	≥36.0 ° C (n=549)	<36.0 ° C (n=24)	≥36.0 ° C (n=230)
Body temperature measurement, n, %							
Bladder	559 51.3%	18 52.9%	82 43.4%	36 57.1%	288 52.5%	15 62.5%	120 52.2%
Axilla	416 38.2%	11 32.4%	82 43.4%	22 34.9%	209 38.1%	6 25.0%	86 37.4%
Tympani	68 6.2%	4 11.8%	17 9.0%	5 7.9%	29 5.3%	2 8.3%	11 4.8%
Rectum	32 2.9%	1 2.9%	2 1.1%		17 3.1%	1 4.2%	11 4.8%
Intravascular	2 0.2%		1 0.5%		1 0.2%		
Non available	12 1.1%		5 2.7%		5 0.9%		2 0.9%
Vasopressor use, n, %							
Any vasopressor	677 62.2%	25 73.5%	116 61.4%	49 77.8%	343 62.5%	14 58.3%	130 56.5%
Noradrenaline	645 59.2%	24 70.6%	111 58.7%	46 73.0%	328 59.7%	14 58.3%	122 53.0%
Dopamine	92 8.5%	2 5.9%	10 5.3%	9 14.3%	51 9.3%	3 12.5%	17 7.4%
Dobutamine	68 6.2%	1 2.9%	7 3.7%	4 6.4%	43 7.8%	2 8.3%	11 4.8%
Adrenaline	20 1.8%	0	2 1.1%	2 3.2%	13 2.4%	0	3 1.3%
Vasopressin	130 12.0%	6 17.7%	21 11.1%	13 20.6%	53 9.7%	4 16.7%	33 14.4%

BMI, body mass index
