# Supplementary material for: Association between low body temperature on admission and in-hospital mortality according to body mass index categories of patients with sepsis
Source: Medicine (Baltimore). 2022 Nov 4;101(44):e31657. doi: 10.1097/MD.0000000000031657 (PMC9646569; doi:10.1097/MD.0000000000031657)
Supplement: Supplementary file 4 [file medi-101-e31657-s004.pdf]

Supplemental Table 4. Patient characteristics according to BMI categories.

	All	Low BMI (n=223)	Normal BMI (n=612)	High BMI (n=254))
Septic shock, n, %	698, 62.3%	147, 65.9%	382, 62.4%	145, 57.1%
Hypotension, n, %	543, 49.9%	120 53.8%	322, 52.6%	101, 39.8%
Hyperlactatemia, n, %	724, 66.5%	152 68.2%	408, 66.7%	164, 64.6%
Acute kidney injury, n, %	408, 37.5%	68 30.5%	217, 35.5%	123, 48.4%
Acute lung injury, n, %	141, 14.4%	15 6.7%	82, 13.4%	44, 17.3%
APACHE2 score	22 (17–29)	22 (17-30)	22 (17-29)	23 (16-30)
SOFA score	9 (6–11)	9 (6-11)	8.5 (6-11)	9 (5-12)

Number of patients with missing data: SOFA score n=158; APACHE II score, n=126
APACHE, acute physiology and chronic health evaluation; BMI, body mass index; SOFA, sequential organ failure assessment

Supplemental Table 5. Patient characteristics with or without low body temperature.

	All	Low BT (n=121)	Non low BT (n=968)
Septic shock, n, %	674, 61.9%	91, 75.2%	583, 60.2%
Hypotension, n, %	543, 49.9%	73, 60.3%	470, 48.6%
Hyperlactatemia, n, %	724, 66.5%	88, 72.7%	636, 65.7%
Acute kidney injury, n, %	408, 37.5%	68, 56.2%	340, 35.1%
Acute lung injury, n, %	141, 14.4%	18, 14.9%	123, 12.7%
APACHE2 score	22 (17–29)	28 (21-34)	22 (16-29)
SOFA score	9 (6–11)	10 (8-13)	8 (5-11)

Number of patients with missing data: SOFA score n=158; APACHE II score, n=126
APACHE, acute physiology and chronic health evaluation; BMI, body mass index; SOFA, sequential organ failure assessment
