# Supplementary material for: Association between low body temperature on admission and in-hospital mortality according to body mass index categories of patients with sepsis
Source: Medicine (Baltimore). 2022 Nov 4;101(44):e31657. doi: 10.1097/MD.0000000000031657 (PMC9646569; doi:10.1097/MD.0000000000031657)
Supplement: Supplementary file 5 [file medi-101-e31657-s005.pdf]

Supplemental Table 6. Odds ratios of factors of worse in-hospital mortality (BMI as a continuous variable)

**Variables**	Odds ratio	95% CI	P value
<36 °C	1.240	0.776–1.980	0.37
Age	1.016	1.003–1.029	0.01
Charlson Comorbidity Index	1.221	1.115–1.336	<.0001
SOFA score	1.178	1.126–1.231	<.0001
BMI	1.008	0.976–1.041	0.63

BMI, body mass index; CI, confidence interval; SOFA, Sequential Organ Failure Assessment
