# Supplementary material for: Association between low body temperature on admission and in-hospital mortality according to body mass index categories of patients with sepsis
Source: Medicine (Baltimore). 2022 Nov 4;101(44):e31657. doi: 10.1097/MD.0000000000031657 (PMC9646569; doi:10.1097/MD.0000000000031657)
Supplement: Supplementary file 6 [file medi-101-e31657-s006.pdf]

Supplemental Table 7. P-value for the interaction for in-hospital mortality (BMI as a continuous variable)

Variables	p-value for interaction
BMI x low BT	0.1403
BMI x normal BT	0.0141
BMI x high BT	0.1260

BMI, body mass index; BT, body temperature
